# Reference-Driven Undersampled MR Image Reconstruction Using Wavelet Sparsity-Constrained Deep Image Prior

**DOI:** 10.1155/2021/8865582

**Published:** 2021-01-20

**Authors:** Di Zhao, Yanhu Huang, Feng Zhao, Binyi Qin, Jincun Zheng

**Affiliations:** ^1^Key Laboratory of Complex System Optimization and Big Data Processing, Guangxi Colleges and Universities, Yulin Normal University, Yulin 537000, China; ^2^School of Physics and Telecommunication Engineering, Yulin Normal University, Yulin 537000, China

## Abstract

Deep learning has shown potential in significantly improving performance for undersampled magnetic resonance (MR) image reconstruction. However, one challenge for the application of deep learning to clinical scenarios is the requirement of large, high-quality patient-based datasets for network training. In this paper, we propose a novel deep learning-based method for undersampled MR image reconstruction that does not require pre-training procedure and pre-training datasets. The proposed reference-driven method using wavelet sparsity-constrained deep image prior (RWS-DIP) is based on the DIP framework and thereby reduces the dependence on datasets. Moreover, RWS-DIP explores and introduces structure and sparsity priors into network learning to improve the efficiency of learning. By employing a high-resolution reference image as the network input, RWS-DIP incorporates structural information into network. RWS-DIP also uses the wavelet sparsity to further enrich the implicit regularization of traditional DIP by formulating the training of network parameters as a constrained optimization problem, which is solved using the alternating direction method of multipliers (ADMM) algorithm. Experiments on *in vivo* MR scans have demonstrated that the RWS-DIP method can reconstruct MR images more accurately and preserve features and textures from undersampled *k*-space measurements.

## 1. Introduction

Magnetic resonance imaging (MRI) is a noninvasive imaging technology that can provide structural, functional, and anatomical information for clinical diagnosis. However, its slow imaging speed may result in motion artifacts and image quality degradation, as well as lead to patient discomfort. To accelerate MRI scans, researchers are seeking methods to increase imaging speed by reducing the amount of acquired *k*-space data without degrading the image reconstruction quality.

Accelerated MR image reconstruction from undersampled *k*-space measurements is, in essence, a highly underdetermined inverse problem. Reconstruction methods based on signal processing have evolved rapidly over the past decades and can now explore and utilize the prior information about the desired MR image to achieve the reconstruction by using regularization methods under the premise of ensuring the uniqueness and stability of the solution. Sparsity is a commonly used prior information with the emerging popularity of Compressed Sensing (CS) theory [1–3], including fixed sparse transform (e.g., wavelet or/and gradient) [4–6] and more flexible adaptive sparse representation (e.g., data-driven tight frame [7] and dictionary learning [8–10]). High-resolution reference images obtained in advance in practical application scenarios can also provide prior information. They can provide structural similarity for the target MR images and obtain more sparse difference images [11–13]. In addition, the structured priors, such as image support information [14–16] and structural sparsity (e.g., group sparsity, block sparsity, and tree sparsity) [15, 17, 18], can be introduced into a reconstruction model based on the union-of-subspace sampling theory [19], which has been verified to be efficient in improving reconstruction accuracy.

In recent years, deep learning has received a great deal of attention in the field of medical imaging, especially for segmentation, denoising, classification, and acceleration of MRI tasks [20]. MRI approaches based on deep learning can be either data-driven or model-driven [21, 22]. Data-driven approaches are aimed at learning the mapping from undersampled *k*-space/images to fully sampled *k*-space/images [23–28]. Model-driven approaches start from MR image reconstruction models and import the procedure of iterative reconstruction algorithms into networks [29–32]. To ensure the quality of reconstruction performance, both approaches require pre-training processes with the aid of large, high-quality patient-based datasets. However, this is a challenge in clinical applications because it is difficult to obtain sufficient amounts of patient-based MR datasets due to patient privacy concerns.

Recently, Ulyanov et al. proposed a Deep Image Prior (DIP) framework [33], which demonstrates that convolutional neural networks (CNNs) have the inherent ability to regularize various ill-posed inverse problems without pretraining [34]. DIP can achieve satisfactory results by applying untrained networks with random noise as the network input. DIP has been used for denoising, inpainting, super-resolution reconstruction [35–38], CS recovery [39], and medical imaging, such as PET image reconstruction [34], CT reconstruction [40], and dynamic MRI [41].

In this paper, we propose a novel deep learning-based Reference-driven method using Wavelet Sparsity-constrained DIP (RWS-DIP) for CS-based undersampled MR image reconstruction, which can achieve improved performance without any pre-training procedures. Our proposed RWS-DIP method incorporates structure and sparsity priors into a DIP framework and utilizes the priors to further improve the efficiency of learning. It not only builds a bridge between the constrained reconstruction method and deep learning, but also largely reduces the dependence on patient-based datasets and contributes to the expansion of clinical applications. Experimental results have shown that the proposed RWS-DIP method can obtain more accurate reconstruction than traditional DIP, particularly in preserving image textures and features. The main contributions of this paper can be summarized as follows:

1. The proposed RWS-DIP method utilizes both structure and sparsity priors of MR images. The former is introduced by using a high-resolution reference image obtained in advance as the input of CNN, whose structure is similar to target MR images and thereby incorporates structural information into network. The latter is used by regularizing the *l*_1_ norm of coefficients in a wavelet domain to further enrich the implicit regularization of traditional DIP, which is enforced by the fixed network structure. These priors improve the efficiency and effectiveness of deep learning and contribute to the improvement in reconstruction performance
2. The proposed RWS-DIP is a novel deep learning-based MR image reconstruction method inspired by traditional DIP and does not require any pre-training. This advantage renders the training datasets unnecessary, which has significance in clinical applications

The remainder of this paper is organized as follows.  Section 2 presents details on the proposed RWS-DIP method, as well as a review of traditional DIP.  Section 3 includes experimental results from *in vivo* MR scans and also includes details about data acquisition, undersampled schemes, and the experimental setup.  Section 4 provides a summary of the paper's main points and its results.

## 2. Methodology

### 2.1. Traditional DIP for Undersampled MR Image Reconstruction

Applying traditional DIP to undersampled MR image reconstruction, the object function is

(1) $$\begin{array}{c} \hat{\theta }=\arg\underset{\theta }{\min}{\left\|y-F_{\text{u}}f\left(\theta \;|\;z\right)\right\|}_{2}^{2}, \end{array}$$

where **y** ∈ *ℂ*^*M*×1^ is the undersampled *k*-space measurements of the desired MR image **I**_t_ ∈ *ℂ*^*N*×*N*^, **F**_u_ denotes an undersampled Fourier transform operator, and ‖∙‖_2_ is the *l*_2_ norm. *f*(*θ* | **z**) is an untrained deep CNN parameterized by *θ*, with the random noise **z** as input.

The desired MR image can then be reconstructed by

(2) $$\begin{array}{c} {\hat{I}}_{\text{t}}=f\left(\hat{\theta }\;|\;z\right). \end{array}$$

The training of the network parameters *θ* is performed by solving the optimization problem in Equation (1) iteratively, which is guided by the attempt to best match the network output to the measurements in *k*-space. In DIP, no pre-training procedure is needed and the network training, or optimizing of network parameters, begins with an untrained CNN initialized randomly.

### 2.2. Proposed Method

**Figure 1:**
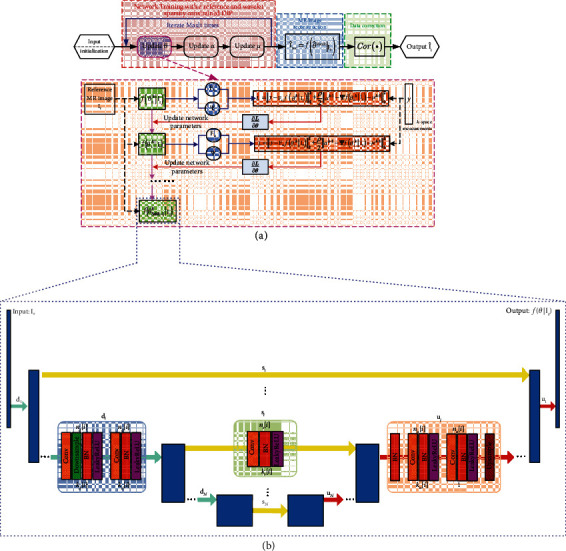
Overview of the proposed RWS-DIP method: (a) overall process for ADMM-based reconstruction; (b) network architecture [33] used in the proposed method.

Figure 1 depicts an overview of our proposed RWS-DIP method, in which the procedure of the target MR image reconstruction can be achieved in three steps: network training, MR image reconstruction, and data correction. In the first step, we do not need high-quality MR datasets and pre-training. The network parameters of the untrained CNN are optimized by solving the proposed constrained object function iteratively, which not only restricts the data consistency and explores wavelet sparsity but also introduces structural prior by using a similar reference image as the input of CNN. Next, the trained network outputs the reconstructed MR image. In the third step, the data correction process uses the prior measurements in *k*-space to further improve the reconstruction accuracy. A further explanation will be provided in the following sections.

#### 2.2.1. Network Training with a Reference and Wavelet Sparsity-Constrained DIP

Leveraging the concept of the traditional DIP framework, our proposed RWS-DIP method uses a high-resolution reference MR image and the wavelet sparsity to provide prior information for the target MR image reconstruction. Therefore, the objective function for network parameter optimization is as follows:

(3) $$\begin{array}{c} \hat{\theta }=\arg\underset{\theta }{\min}{\left\|y-F_{\text{u}}f\left(\theta \;|\;I_{\text{r}}\right)\right\|}_{2}^{2}+\lambda {\left\|\Psi f\left(\theta \;|\;I_{\text{r}}\right)\right\|}_{1}, \end{array}$$

where **I**_r_ ∈ *ℂ*^*N*×*N*^ denote a high-resolution reference MR image acquired in advance with similar anatomical structure to the target image **I**_t_ ∈ *ℂ*^*N*×*N*^, Ψ is the wavelet transform operator, and ‖∙‖_1_ is the *l*_1_ norm. The regularization parameter *λ* > 0.

Our proposed objective function in Equation (3) consists of the data fidelity term and the *l*_1_ regularization term. It is aimed at finding the optimal network parameters that ensure the sparsity of the target MR image in wavelet domain on the premise of maintaining data consistency.

The data fidelity term restricts the data consistency between the network output and *k*-space measurements. We use the known reference MR image **I**_r_ as the network input, instead of random noise in traditional DIP. This strategy is capable of exploring and introducing the structural prior of the target MR image into the network for learning because of the high structural similarity between the reference and target images. The *l*_1_ regularization constrains the sparsity of the target MR image in a wavelet domain, which merges more prior information for efficient training of network parameters.

Let *α* = Ψ*f*(*θ* | **I**_r_), Equation (3) becomes

(4) $$\begin{array}{c} \hat{\theta }=\text{argmin}{\left\|y-F_{\text{u}}f\left(\theta \;|\;I_{\text{r}}\right)\right\|}_{2}^{2}+\lambda {\left\|\alpha \right\|}_{1}\\ \text{s}.\text{t}.\;\alpha =\Psi f\left(\theta \;|\;I_{\text{r}}\right). \end{array}$$

The constrained optimization problem in Equation (4) can be transformed into a penalty using the augmented Lagrangian:

(5) $$\begin{array}{c} \arg\underset{\theta ,\alpha }{\min}{\left\|y-F_{\text{u}}f\left(\theta \;|\;I_{\text{r}}\right)\right\|}_{2}^{2}+\lambda {\left\|\alpha \right\|}_{1}+\frac{\rho }{2}{\left\|\alpha -\Psi f\left(\theta \;|\;I_{\text{r}}\right)-\mu \right\|}_{2}^{2}. \end{array}$$

In the expression above, *μ* stands for the Lagrange multiplier vector and *ρ* is a penalty parameter.

To solve the problem in Equation (5), we use the alternating direction method of multipliers (ADMM) algorithm [42] to update the three unknowns *θ*, *α*, and *μ* iteratively:

(6) $$\begin{array}{c} {\hat{\theta }}^{k}=\arg\underset{\theta }{\min}{\left\|y-F_{\text{u}}f\left(\theta \;|\;I_{\text{r}}\right)\right\|}_{2}^{2}+\frac{\rho }{2}{\left\|\alpha^{k-1}-\Psi f\left(\theta \;|\;I_{\text{r}}\right)-\mu^{k-1}\right\|}_{2}^{2}, \end{array}$$

(7) $$\begin{array}{c} \alpha^{k}=\arg\underset{\alpha }{\min}\lambda {\left\|\alpha \right\|}_{1}+\frac{\rho }{2}{\left\|\alpha -\Psi f\left({\hat{\theta }}^{k}\;|\;I_{\text{r}}\right)-\mu^{k-1}\right\|}_{2}^{2}, \end{array}$$

(8) $$\begin{array}{c} \mu^{k}=\mu^{k-1}+\alpha^{k}-\Psi f\left({\hat{\theta }}^{k}\;|\;I_{\text{r}}\right). \end{array}$$

(1) For the subproblem in Equation (6), this optimization is close in spirit to that performed in traditional DIP. However, we further modify the optimization by a proximity regularization that forces Ψ*f*(*θ* | **I**_r_) to be close to (*α*^*k*−1^ − *μ*^*k*−1^), which helps to provide additional stabilization and robustness
(2) For the subproblem in Equation (7), the solution can be written as
  (9) $$\begin{array}{c} \alpha^{k}={\text{S}}_{\frac{\lambda }{\rho }}\left(\Psi f\left({\hat{\theta }}^{k}\;|\;I_{\text{r}}\right)+\mu^{k-1}\right), \end{array}$$
  where S_*λ*/*ρ*_ is the soft thresholding operator defined as [42]
  (10) $$\begin{array}{c} S_{\kappa }\left(b\right)=\left\{\begin{array}{cc} b-\kappa , & b>\kappa ,\\ 0, & \left|b\right|\leq \kappa ,\\ b+\kappa , & b<-\kappa \end{array}\right. \end{array}$$

#### 2.2.2. MR Image Reconstruction

After the iterative update procedure of network parameters, we obtain the trained CNN parameterized by ${\hat{\theta }}^{\text{MaxIt}}$(let **MaxIt** denote the maximum iteration number of ADMM; then, ${\hat{\theta }}^{\text{MaxIt}}$ is the parameter of the final trained network). The output of the trained CNN is the reconstructed MR image, which can be presented as

(11) $$\begin{array}{c} {\hat{I}}_{\text{rec}}=f\left({\hat{\theta }}^{\text{MaxIt}}\;|\;I_{\text{r}}\right). \end{array}$$

#### 2.2.3. Data Correction

Performing data correction operator Cor(·) to CNN output ${\hat{I}}_{\text{rec}}$ in the last step below, we obtain corrected *k*-space data **y**_cor_ as follows:

(12) $$\begin{array}{c} y_{\text{cor}}=\text{Cor}\left({\hat{I}}_{\text{rec}}\right)={\left(F{\hat{I}}_{\text{rec}}\right)}_{\overset{¯}{\text{U}}}⋃\;\;y, \end{array}$$

where **F** denotes Fourier transform and **y** is the priori acquired measurements of the target MR image, which are sampled at the spatial locations corresponding to the undersampled mask U in *k*-space. Let $\overset{¯}{\text{U}}$ denote the complementary set of U. This data correction strategy, defined in Equation (12), reserves all the priori acquired measurements to enforce the *k*-space data consistency, so that the reconstruction error will focus only on the missing *k*-space data. The final reconstructed target MR image can then be achieved by performing an inverse Fourier transform on **y**_cor_

(13) $$\begin{array}{c} {\hat{I}}_{\text{t}}=F^{-1}\left(y_{\text{cor}}\right). \end{array}$$

The algorithm flowchart of our proposed RWS-DIP method is presented in Algorithm 1.

### 2.3. Network Architecture

The CNN architecture employed in the proposed RWS-DIP method is summarized in Figure 1(b), which is the same as that used in [33]. It is an encoder-decoder (“hourglass”) architecture with skip connection. The encoding path (left side) and decoding path (right side) are linked by the skip connections, marked by yellow arrows, to integrate features from different resolutions. The network consists of repetitive applications of the convolutional (Conv) layer, batch normalization (BN) layer, and leaky rectified linear unit (LeakyReLU) layer, downsampling with stride and upsampling with bilinear interpolation. The maximal depth of the network is *L*. *n*_*d*_[*i*], *n*_*u*_[*i*], and *n*_*s*_[*i*] denote the number of filters at the *i*th depth for downsampling, upsampling, and skip connections, respectively. *k*_*d*_[*i*], *k*_*u*_[*i*], and *k*_*s*_[*i*] correspond to the respective kernel sizes.

## 3. Experimental Results

### 3.1. Experimental Setup

Experiments were conducted to evaluate the performance of our proposed RWS-DIP method. The comparisons with the proposed RWS-DIP method included zero-filling and traditional DIP [33]. To ensure a fair comparison, the zero-filling reconstructions and corresponding *k*-space measurements were used as inputs for all the methods, and the same network architectures was employed for our RWS-DIP method and traditional DIP.

We quantified the reconstruction quality using the metrics of relative error (RelErr), peak signal-to-noise ratio (PSNR), and structural similarity index (SSIM) [43]:

(14) $$\begin{array}{c} \text{RelErr}=\frac{{\left\|\hat{x}-x\right\|}_{2}}{{\left\|x\right\|}_{2}}, \end{array}$$

(15) $$\begin{array}{c} \text{PSNR}=10\mathrm{lg}\frac{NN{\left(\text{MA}{\text{X}}_{x}\right)}^{2}}{\sum_{i=1}^{N}\;\;\sum_{j=1}^{N}\;\;\left[\hat{x}\left(i,j\right)-x\left(i,j\right)\right]}, \end{array}$$

(16) $$\begin{array}{c} \text{SSIM}=\frac{\left(2\mu_{x}\mu_{\hat{x}}+c_{1}\right)\left(2\sigma_{x\hat{x}}+c_{2}\right)}{\left(\mu_{x}^{2}+\mu_{\hat{x}}^{2}+c_{1}\right)\left(\sigma_{x}^{2}+\sigma_{\hat{x}}^{2}+c_{2}\right)}. \end{array}$$

In the descriptions in Equations (14)–(16), the reconstructed MR image $\hat{x}$ and the ground truth **x** are the same size of *N* × *N*, and MAX_**x**_ denotes the largest value in **x**. Moreover, for the SSIM shown in Equation (16), *μ*_**x**_, $\mu_{\hat{x}}$, *σ*_**x**_, and $\sigma_{\hat{x}}$ represent the means and standard deviations of **x** and $\hat{x}$, respectively, and $\sigma_{x\hat{x}}$ denotes the crosscovariance between **x** and $\hat{x}$, and constants *c*_1_ = 0.01 and *c*_2_ = 0.03.

#### 3.1.1. Data Acquisition

**Figure 2:**
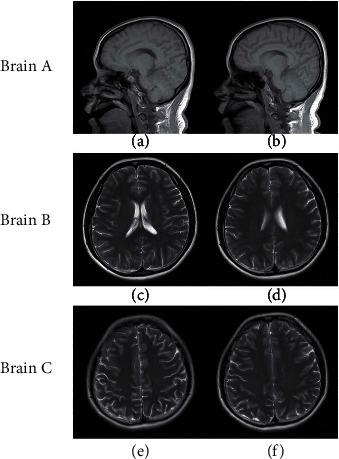
MR images used in the experiments: Brain A: the reference image (a) and target image (b); Brain B: the reference image (c) and target image (d); Brain C: the reference image (e) and target image (f).

To demonstrate the performance of our RWS-DIP method, simulations were conducted on three groups of in*vivo* MR images. To simulate the data acquisition, we undersampled the 2D discrete Fourier transform of the MR images from in*vivo* MR scans, which were acquired from a 3T Siemens MRI scanner. The imaging parameters of the first group of scanned data (Brain A) were GR sequence, flip angle = 70°, TR = 250 ms, TE = 2.5 ms, field of view (FOV) = 220 mm × 220 mm, and slice thickness = 5.0 mm. The reference and target images in Brain A were of size 512 × 512, as shown in Figures 2(a) and 2(b). The imaging parameters of the second and third groups of scanned data (Brain B and Brain C) were as follows: SE sequence, flip angle = 120°, TR = 4000 ms, TE = 91 ms, FOV = 176 mm × 176 mm, and slice thickness = 5.0 mm. The MR images in Brain B and Brain C were of size 256 × 256 and are shown in Figures 2(c)–2(f), respectively.

#### 3.1.2. Training Setting

We used the same CNN architecture as the traditional DIP in [33], which is shown in detail in Figure 1(b). The parameters used in the experiments, including network hyperparameters, iteration number (MaxIt and SubIt), wavelet (wavelet function and decomposition level), ADMM penalty parameter *ρ*, and regularization parameter *λ*, are shown in Table 1.

The models were implemented on the Ubuntu 16.04 LTS (64 bit) operating system, running on an Intel Core i9-7920X 2.9 GHz CPU and Nvidia GeForce GTX 1080Ti GPU with 11 GB RAM in the PyTorch open framework with CUDA and CUDNN support.

#### 3.1.3. Undersampled Schemes

**Figure 3:**
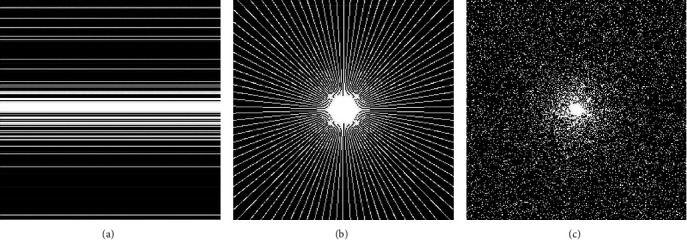
Undersampling masks used in the experiments: (a) Cartesian mask with a sampling rate of 20%; (b) radial mask with a sampling rate of 20%; (c) variable density mask with sampling rate of 15%.

To compare the influence of different undersampling masks to the performance of the proposed RWS-DIP method, our experiments employed three types of undersampling masks: Cartesian, variable density, and radial.  Figure 3 depicts these three undersampling masks.

### 3.2. Results

#### 3.2.1. Reconstruction Performance Comparison

*(1) Reconstruction under Different Sampling Rates*. We demonstrated the effectiveness of our RWS-DIP method at different sampling rates under Cartesian mask.  Table 2 shows the quantitative performance of the proposed RWS-DIP method, traditional DIP and zero-filling reconstructions in RelErr, and PSNR and SSIM indexes at 10%, 20%, 30%, and 40% sampling rates. Taking into account the randomness involved in the training procedure (random initialization of network parameters in the proposed method; both random initializations of the network input and network parameters for traditional DIP), all the quantitative results were achieved by averaging the indices after being run 10 times. It can be seen that the proposed method has the lowest RelErr and the highest PSNR and SSIM values for all three groups of MR data, which means that our proposed RWS-DIP method can obtain more accurate reconstruction.

**Figure 4:**
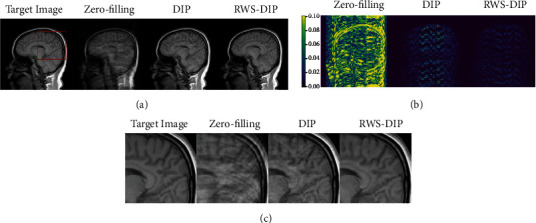
Comparison of reconstructions of the target MR image in Brain A using Cartesian undersampled mask with 20% sampling rate: (a) the target image and reconstruction results, (b) the corresponding error images, and (c) the corresponding zoom-in images.

**Figure 5:**
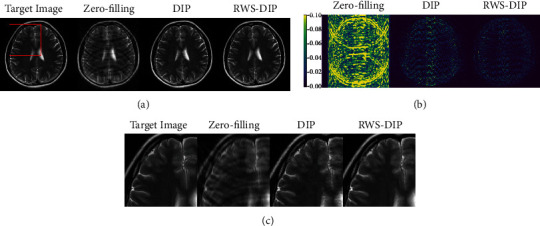
Comparison of reconstructions of the target MR image in Brain B using Cartesian undersampled mask with 30% sampling rate: (a) the target image and reconstruction results, (b) the corresponding error images, and (c) the corresponding zoom-in images.

**Figure 6:**
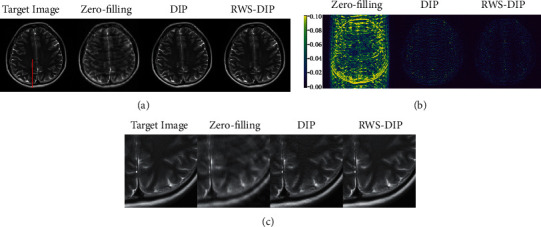
Comparison of reconstructions of the target MR image in Brain C using Cartesian undersampled mask with 30% sampling rate: (a) the target image and reconstruction results, (b) the corresponding error images, and (c) the corresponding zoom-in images.

Figures 4–6 show the reconstructed MR images using the proposed RWS-DIP method and the compared methods under Cartesian undersampled mask with 20% and 30% sampling rates. It is obvious that our RWS-DIP method has the best performance in preserving more image textures and features, especially from the zoom-in images. The corresponding error images further show that the reconstruction of our RWS-DIP method has the smallest differences and is closest to the target MR image.

**Figure 7:**
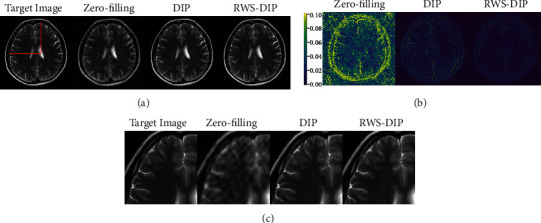
Comparison of reconstructions of the target MR image in Brain B using the radial undersampled mask with 20% sampling rate: (a) the target image and reconstruction results, (b) the corresponding error images, and (c) the corresponding zoom-in images.

**Figure 8:**
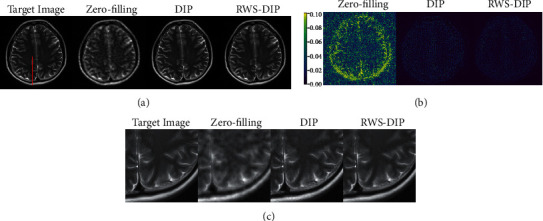
Comparison of reconstructions of the target MR image in Brain C using the variable density undersampled mask with 30% sampling rate: (a) the target image and reconstruction results, (b) the corresponding error images, and (c) the corresponding zoom-in images.

*(2) Reconstruction with Different Undersampled Masks*. The reconstruction results were compared under radial and variable density undersampled masks. The quantitative results tabulated in Table 3 clearly indicate that the proposed RWS-DIP method obtains more accurate reconstruction than with the radial and variable density undersampled masks. Comparisons of the reconstructed MR images are shown in Figures 7 and 8. The corresponding error images and zoom-in images demonstrate that our RWS-DIP method outperforms the compared methods with less structural loss and can preserve more details than the radial and variable density undersampled masks.

#### 3.2.2. Convergence Analysis

**Figure 9:**
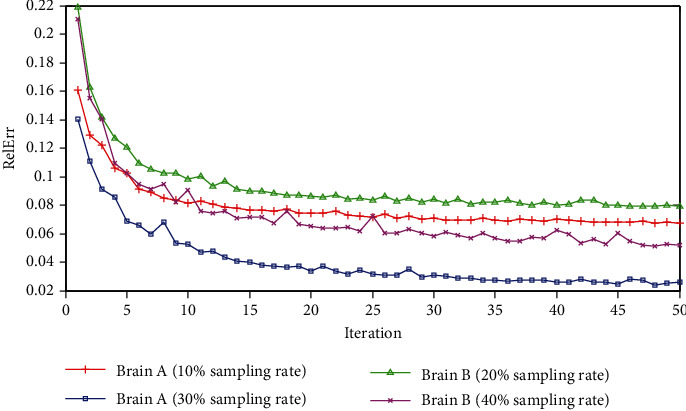
RelErr curves of the proposed RWS-DIP method under Cartesian undersampled mask.

Convergence is an important quality in applications of MRI methods based on deep learning. Therefore, we detected the convergence of the proposed RWS-DIP method use error curves drawn by conducting experiments on Brain A and Brain B under Cartesian undersampled mask.  Figure 9 depicts the relative errors of reconstruction at every ADMM iteration. It can be observed that, as the number of iterations increases, the relative errors gradually converge to a low value at different sampling rates. Although there are slight fluctuations in the iteration procedure, the overall trend maintains convergence.

#### 3.2.3. Parameter Evaluation

We evaluated the sensitivity of the proposed RWS-DIP method to parameter settings. The main parameters evaluated were the ADMM penalty parameter *ρ* and the regularization parameter *λ*. We performed experiments on the Brain C dataset under Cartesian undersampled mask and varied one parameter at a time while keeping the rest as fixed values, as shown in Table 1.

**Figure 10:**
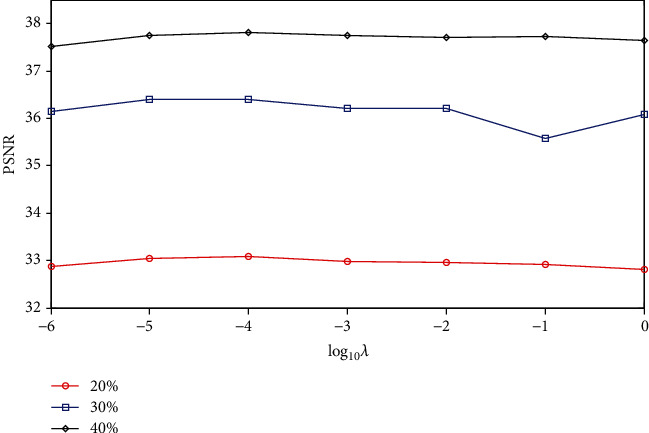
PSNR values vs. regularization parameter *λ* for the reconstruction under Cartesian undersampled mask with different sampling rates.

**Figure 11:**
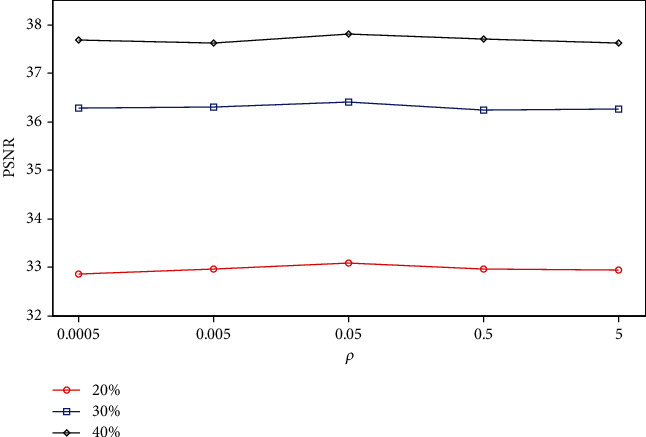
PSNR values vs. ADMM penalty parameter *ρ* for the reconstruction under Cartesian undersampled mask with different sampling rates.

Figures 10 and 11 show the plots of PSNR values as a function of the ADMM penalty parameter *ρ* and the regularization parameter *λ*. As can be seen from the curves, the optimal numerical settings for *ρ* and *λ* (*ρ* = 0.05 and *λ* = 0.0001) in the proposed RWS-DIP method under different sampling rates are identical, which means that the RWS-DIP method has robustness in the setting of parameters. In fact, although the reconstructions have lower PSNR values than other numerical settings for parameters *ρ* and *λ*, the difference is not significant, and the reconstruction performance is acceptable.

## 4. Conclusions

In this paper, we propose a novel reference-driven undersampled MR image reconstruction method using wavelet sparsity-constrained deep image prior. Our RWS-DIP method, which is based on the DIP framework, requires neither a pre-training procedure nor patient-based datasets, which is of great significance for clinical applications. The RWS-DIP method uses both structure and sparsity priors to improve the efficiency of the learning. The structural prior is introduced by employing a reference image as the network input, and the sparsity prior is explored by regularizing the *l*_1_ norm of wavelet coefficients. Experimental results on in*vivo* MR scans show that the RWS-DIP method can achieve improved reconstruction performance and outperforms traditional DIP in preserving texture details and removing artifacts.

Two extensions can be made in order to improve the proposed scheme: (1) mining and incorporating more effective prior information may lead to a further boost in performance, particularly in regard to strengthening the use of structural prior information, and (2) further research is needed for the regularization effect introduced into DIP, which will guide the design of complementary regularizations, so as to achieve a stronger effect and better performance.

## Figures and Tables

**Algorithm 1:**
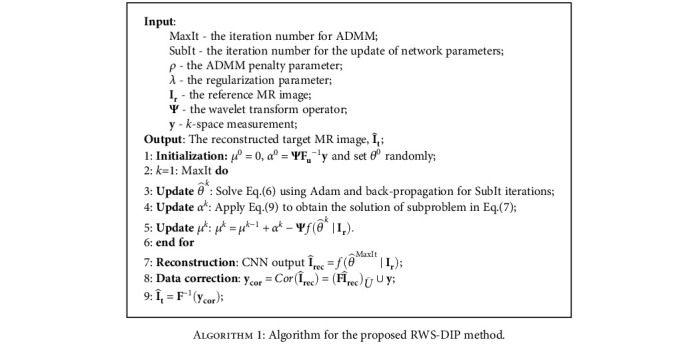
Algorithm for the proposed RWS-DIP method.

**Table 1:** Parameter settings for experiments.

Parameter		Images		
		Brain A	Brain B	Brain C
Network hyperparameters	Learning rate	0.01	0.01	0.01
	*L*	6	6	6
	*n*_*d*_	[16, 32, 64, 64, 128, 128]	[32, 32, 64, 128, 128, 128]	[32, 32, 64, 128, 128, 128]
	*n*_*u*_	[16, 32, 64, 64, 128, 128]	[32, 32, 64, 128, 128, 128]	[32, 32, 64, 128, 128, 128]
	*n*_*s*_	[16,16,16,16,16,16]	[16,16,16,16,16,16]	[16,16,16,16,16,16]
	*k*_*d*_	[3,3,3,3,3,3]	[3,3,3,3,3,3]	[3,3,3,3,3,3]
	*k*_*u*_	[3,3,3,3,3,3]	[3,3,3,3,3,3]	[3,3,3,3,3,3]
	*k*_*s*_	[1,1,1,1,1,1]	[1,1,1,1,1,1]	[1,1,1,1,1,1]

Iteration number	MaxIt	50	50	50
	SubIt	100	100	100

Wavelet parameters	Wavelet function	Haar	Haar	Haar
	Decomposition level	8	6	6

*ρ*		0.07	0.05	0.05

*λ*		0.0001	0.0001	0.0001

**Table 2:** RelErr, PSNR, and SSIM values of reconstruction by different methods under Cartesian undersampled mask.

Images	Methods		10%			20%	
		RelErr (%)	PSNR (dB)	SSIM	RelErr (%)	PSNR (dB)	SSIM

Brain A	Zero-filling	21.63	21.6857	0.7101	15.26	24.7174	0.7695
	DIP	16.49	24.1475	0.8263	5.45	33.6852	0.9617
	RWS-DIP	6.92	31.5838	0.9486	3.21	38.2738	0.9836

Brain B	Zero-filling	35.26	20.2926	0.6391	18.74	25.7849	0.7608
	DIP	33.08	20.8466	0.7212	11.31	30.1983	0.9361
	RWS-DIP	15.96	27.1810	0.9000	7.59	33.6347	0.9694

Brain C	Zero-filling	32.53	21.3240	0.6600	15.99	27.4915	0.7860
	DIP	30.78	21.8126	0.7353	11.74	30.1815	0.9297
	RWS-DIP	18.09	26.4293	0.8744	8.41	33.0789	0.9635

Images	Methods		30%			40%	
		RelErr (%)	PSNR (dB)	SSIM	RelErr (%)	PSNR (dB)	SSIM

Brain A	Zero-filling	5.39	33.7439	0.8430	4.02	36.3000	0.8590
	DIP	2.82	39.4789	0.9862	2.54	40.4871	0.9876
	RWS-DIP	2.01	42.3201	0.9917	1.67	43.9822	0.9942

Brain B	Zero-filling	16.80	26.7302	0.7699	8.89	32.2654	0.8302
	DIP	8.38	32.7954	0.9593	6.34	35.2549	0.9733
	RWS-DIP	5.73	36.0731	0.9795	4.35	38.4773	0.9864

Brain C	Zero-filling	11.03	30.7196	0.8346	7.94	33.5719	0.8597
	DIP	7.21	34.4198	0.9698	6.31	35.5848	0.9747
	RWS-DIP	5.73	36.4088	0.9808	4.88	37.8079	0.9845

**Table 3:** RelErr, PSNR, and SSIM values of reconstruction by different methods under radial undersampled mask and variable density undersampled mask.

Images	Mask (undersampled rate)	Methods	RelErr (%)	PSNR (dB)	SSIM
Brain A	Radial (10%)	Zero-filling	10.15	28.2588	0.7601
		DIP	6.25	33.6669	0.9408
		RWS-DIP	3.52	37.4635	0.9780
	Variable density (20%)	Zero-filling	7.93	30.3949	0.8483
		DIP	3.35	38.0061	0.9798
		RWS-DIP	2.57	40.2062	0.9859

Brain B	Radial (20%)	Zero-filling	15.35	27.5173	0.7928
		DIP	8.20	32.9691	0.9610
		RWS-DIP	5.76	36.0310	0.9786
	Variable density (30%)	Zero-filling	16.99	26.6374	0.7596
		DIP	6.38	35.1479	0.9708
		RWS-DIP	4.75	37.7008	0.9827

Brain C	Radial (20%)	Zero-filling	12.80	29.4250	0.8256
		DIP	8.04	33.4771	0.9623
		RWS-DIP	6.02	35.9762	0.9775
	Variable density (30%)	Zero-filling	14.34	28.4345	0.8038
		DIP	7.03	34.6578	0.9692
		RWS-DIP	5.18	37.2897	0.9811

## Data Availability

The data used to support the findings of this study are available from the corresponding author on reasonable request.

## References

[1] Donoho D. L. (2006). Compressed sensing. *IEEE Transactions on Information Theory*.

[2] Candès E. J., Romberg J. K., Tao T. (2006). Stable signal recovery from incomplete and inaccurate measurements. *Communications on Pure and Applied Mathematics*.

[3] Davenport M. A., Duarte M. F., Eldar Y. C., Kutyniok G. (2012). *Introduction to Compressed Sensing, Compressed Sensing: Theory and Applications*.

[4] Lustig M., Donoho D., Pauly J. M. (2007). Sparse MRI: the application of compressed sensing for rapid MR imaging. *Magnetic Resonance in Medicine*.

[5] Kim Y., Altbach M. I., Trouard T. P., Bilgin A. Compressed sensing using dual-tree complex wavelet transform.

[6] Qu X., Guo D., Ning B. (2012). Undersampled MRI reconstruction with patch-based directional wavelets. *Magnetic Resonance Imaging*.

[7] Liu J., Wang S., Peng X., Liang D. (2015). Undersampled MR image reconstruction with data-driven tight frame. *Computational and Mathematical Methods in Medicine*.

[8] Zhan Z., Cai J. F., Guo D., Liu Y., Chen Z., Qu X. (2016). Fast multiclass dictionaries learning with geometrical directions in MRI reconstruction. *IEEE Transactions on Biomedical Engineering*.

[9] Liu Q., Wang S., Ying L., Peng X., Zhu Y., Liang D. (2013). Adaptive dictionary learning in sparse gradient domain for image recovery. *IEEE Transactions on Image Processing*.

[10] Ophir B., Lustig M., Elad M. (2011). Multi-scale dictionary learning using wavelets. *IEEE Journal of Selected Topics in Signal Processing*.

[11] Du H., Lam F. (2012). Compressed sensing MR image reconstruction using a motion-compensated reference. *Magnetic Resonance Imaging*.

[12] Peng X., Du H. Q., Lam F., Babacan D., Liang Z. P. Reference driven MR image reconstruction with sparsity and support constraints.

[13] Lam F., Haldar J. P., Liang Z. P. Motion compensation for reference-constrained image reconstruction from limited data.

[14] Manduca A., Trzasko J. D., Li Z. Compressive sensing of images with a priori known spatial support.

[15] Han Y., du H., Gao X., Mei W. (2017). MR image reconstruction using cosupport constraints and group sparsity regularisation. *IET Image Processing*.

[16] Wang Y., Zhao D., Ma S. L., Du H. Q. Mr image reconstruction from undersampled measurements using union-of-subspaces.

[17] Stojnic M., Parvaresh F., Hassibi B. (2009). On the reconstruction of block-sparse signals with an optimal number of measurements. *IEEE Transactions on Signal Processing*.

[18] Usman M., Prieto C., Schaeffter T., Batchelor P. G. (2011). k-t group sparse: a method for accelerating dynamic MRI. *Magnetic Resonance in Medicine*.

[19] Blumensath T. (2011). Sampling and reconstructing signals from a union of linear subspaces. *IEEE Transactions on Information Theory*.

[20] Litjens G., Kooi T., Bejnordi B. E. (2017). A survey on deep learning in medical image analysis. *Medical Image Analysis*.

[21] Cheng J., Wang H. F., Zhu Y. J. (2019). Model-based deep medical imaging: the roadmap of generalizing iterative reconstruction model using deep learning. https://arxiv.org/abs/1906.08143.

[22] Liang D., Cheng J., Ke Z., Ying L. (2020). Deep magnetic resonance image reconstruction: inverse problems meet neural networks. *IEEE Signal Processing Magazine*.

[23] Eo T., Jun Y., Kim T., Jang J., Lee H. J., Hwang D. (2018). Kiki-net: cross-domain convolutional neural networks for reconstructing undersampled magnetic resonance images. *Magnetic Resonance in Medicine*.

[24] Wang S. S., Su Z. H., Ying L. Accelerating magnetic resonance imaging via deep learning.

[25] Yang G., Yu S., Dong H. (2018). Dagan: deep de-aliasing generative adversarial networks for fast compressed sensing MRI reconstruction. *IEEE Transactions on Medical Imaging*.

[26] Schlemper J., Caballero J., Hajnal J. V., Price A. N., Rueckert D. (2018). A deep cascade of convolutional neural networks for dynamic MR image reconstruction. *IEEE Transactions on Medical Imaging*.

[27] Quan T. M., Nguyen-Duc T., Jeong W. K. (2018). Compressed sensing MRI reconstruction using a generative adversarial network with a cyclic loss. *IEEE Transactions on Medical Imaging*.

[28] Akcakaya M., Moeller S., Weingartner S., Ugurbil K. (2019). Scan-specific robust artificial-neural-networks for *k*-space interpolation (RAKI) reconstruction: database-free deep learning for fast imaging. *Magnetic Resonance in Medicine*.

[29] Aggarwal H. K., Mani M. P., Jacob M. (2019). Modl: model-based deep learning architecture for inverse problems. *IEEE Transactions on Medical Imaging*.

[30] Yang Y., Sun J., Li H. B., Xu Z. B. Deep ADMM-Net for compressive sensing MRI.

[31] Qin C., Schlemper J., Caballero J., Price A. N., Hajnal J. V., Rueckert D. (2019). Convolutional recurrent neural networks for dynamic MR image reconstruction. *IEEE Transactions on Medical Imaging*.

[32] Hammernik K., Klatzer T., Kobler E. (2018). Learning a variational network for reconstruction of accelerated MRI data. *Magnetic Resonance in Medicine*.

[33] Ulyanov D., Vedaldi A., Lempitsky V. (2017). Deep image prior. https://arxiv.org/abs/1711.10925v3.

[34] Gong K., Catana C., Qi J., Li Q. (2019). Pet image reconstruction using deep image prior. *IEEE Transactions on Medical Imaging*.

[35] Mataev G., Elad M., Milanfar P. (2019). Deep red: deep image prior powered by red. https://arxiv.org/abs/1903.10176.

[36] Sagel A., Roumy A., Guillemot C. Sub-dip: optimization on a subspace with deep image prior regularization and application to superresolution.

[37] Liu J. M., Sun Y., Xu X. J., Kamilov U. S. (2018). Image restoration using total variation regularized deep image prior. https://arxiv.org/abs/1810.12864.

[38] Hashimoto F., Ohba H., Ote K., Teramoto A., Tsukada H. (2019). Dynamic pet image denoising using deep convolutional neural networks without prior training datasets. *IEEE Access*.

[39] Veen D. V., Jalal A., Soltanolkotabi M., Price E., Vishwanath S., Dimakis A. G. (2018). Compressed sensing with deep image prior and learned regularization. https://arxiv.org/abs/1806.0643.

[40] Baguer D. O., Leuschner J., Schmidt M. (2020). Computed tomography reconstruction using deep image prior and learned reconstruction methods. https://arxiv.org/abs/2003.04989.

[41] Jin K. H., Gupta H., Yerly J., Stuber M., Unser M. (2019). Time-dependent deep image prior for dynamic MRI. https://arxiv.org/abs/1910.01684v1.

[42] Boyd S., Parikh N., Chu E., Peleato B., Eckstein J. (2010). Distributed optimization and statistical learning via the alternating direction method of multipliers. *Foundations and Trends® in Machine Learning*.

[43] Wang Z., Bovik A. C., Sheikh H. R., Simoncelli E. P. (2004). Image quality assessment: from error visibility to structural similarity. *IEEE Transactions on Image Processing*.

